# *Undaria* marching on; late arrival in the Republic of Ireland

**DOI:** 10.1007/s10811-016-0985-2

**Published:** 2016-10-26

**Authors:** Stefan Kraan

**Affiliations:** Ocean Harvest Technology, Milltown Business Park, Milltown, Co., Galway, H54 D722 Ireland

**Keywords:** Ecological impact, Economic implications, Invasive, Republic of Ireland, Seaweed cultivation, *Undaria pinnatifida*, Wakame

## Abstract

The Asian invasive brown seaweed *Undaria pinnatifida* was found for the first time in the Republic of Ireland in Kilmore Quay in Co. Wexford in July 2016. As this brown kelp is of considerable economic importance and is cultivated in Asia as well as in Europe, it opens up the discussion if this invasive species is socially acceptable to be cultivated in the Republic of Ireland for food and other purposes. This paper briefly examines the global economic importance, cultivation aspects compared to the European native equivalents such as *Alaria esculenta* and *Saccharina latissima*, cultivation yield, economic considerations and the ecological impact of the spread of *Undaria* into non-native areas. Based on the information and facts presented, it is concluded that *Undaria* from a physical, social and economic point of view can be cultivated in Ireland.

## Introduction

*Undaria pinnatifida* (Harvey) Suringar is a brown seaweed of the family Alariaceae Setchell & N.L.Gardner, a kelp indigenous to the Pacific where it is harvested from the wild as well as being cultivated in large quantities for human food applications. In Japan, Korea and China, many tens of thousands of hectares of coastline are devoted to the farming of *Undaria* employing over tens of thousands people from coastal communities in seaweed aquaculture. *Undaria* is widely sold in Asia as a sea vegetable by its trade name Wakame and is used processed in many other foods and snacks (Critchley and Ohno 1998; Prabhasankar et al., 2009). Currently, well over two million tonnes are cultivated worth over US$ 1 billion to the seaweed industry (FAO 2016).

In NW Europe, there is a similar type of kelp from the same family Alariaceae, i.e. *Alaria esculenta* (L.) Greville for which the author coined the phrase “Atlantic Wakame” in the late 1990s. After performing large-scale cultivation and hybridisation experiments in 1999 (Kraan et al. 2000), it was evident that *Alaria* and *Undaria* were very similar in taste and cooking applications. Atlantic Wakame has similar uses, taste and nutritional value as Wakame, although the polysaccharide makeup is different (Schiener et al. 2015). However, the European market for *Alaria* is a fraction of the Asian market for *Undaria*, mainly caused by two factors, price and education on product and usage. Nevertheless, seaweed use and applications is a growing market in Europe and cultivation will be a key aspect of that market in order to control quality and quantity (Yamanaka and Akiyama 1993; Schiener et al. 2015). In the near future, the domestic and European market will increase considerably as the benefits of seaweed as nutritious and health food become more widely known (Holdt and Kraan 2011).

## Dispersal means

*Undaria* inhabits cold temperate coastal waters and grows best at 10–15 °C (Akiyama 1965). Sporophytes are reported to degrade at temperatures above 20 °C and die at temperatures greater than 23 °C (Wallentinus 2007). *Undaria* grows in a wide range of wave exposures from sheltered marinas to the open coast and extends vertically from the low intertidal to 18 m depth and can grow on any hard surface including artificial substrates (although it is most common between 1 and 3 m depth; Minchin and Nunn 2014). *Undaria* is a highly successful species and invader as it grows rapidly and outcompetes many species; can reproduce after 50 days; can probably reproduce all year round and ejects millions of spores which can be transported by currents or lie dormant (for years) until conditions are suitable for growth (Wallentinus 2007). (Nyberg and Wallentinus 2005) ranked specific traits of 113 seaweeds introduced into Europe for the three main categories: dispersal, establishment and ecological impact. *Undaria* ranked third overall as the most invasive seaweed. Fouling appears to be an effective method of dispersing *Undaria*. Examples of this include both the microscopic gametophytes and the sporophytes remaining attached to the hull of boats and yachts after sailing hundreds of kilometres (Ribera and Boudouresque 1995; Schaffelke et al. 2006).

## *Undaria* in Europe

In 1971, *U. pinnatifida* was accidentally introduced into Europe from Asia in the Thau lagoon (Mediterranean France), via oyster spat from Japan (Ribera and Boudouresque 1995). It was then introduced to the French Atlantic coast in 1983 for commercially cultivating the species for the export market (Floc’h et al., 1991). Although it was believed that *Undaria* could not reproduce under the conditions of the French Atlantic coast, it proved scientists wrong. Shortly after its introduction, the seaweed had escaped from the seaweed farm and was colonizing areas of bays and harbours in Brittany causing some nuisance by its clogging of marinas, navigation channels and power station intakes. In northern Spain, *U. pinnatifida* was reported from Ria Ariosa in 1990 (Santiago Caamaño et al. 1990). Through farming, it was later spread along the northern Spanish Atlantic coast down to the Portuguese border, and so its arrival in Portugal in 2008 was to be expected (Minchin and Nunn 2014). *Undaria* had been found attached to floating pontoons in the Solent region of the south coast of England in 1995, most probably introduced by yachts that were the cross channel vector (Fletcher and Manfredi 1995), spreading later to other sites on the south coast and was also detected in the Channel Islands (Eno et al. 1997). In 1999, it arrived in The Netherlands and Belgium (Stegenga 1999; Dumoulin and De Blauwe, 1999; Leliaert et al. 2000).

Latest records date from 2012 where it was found in Northern Ireland in the marina of Carrickfergus, Belfast Lough (Minchin and Nunn 2014), which currently is the most northerly distributed population, but it is postulated that Scotland or southern Norway are the next places of colonization.

The coastal conditions of Ireland are suitable for growth of the species, e.g. the temperature of Asian waters where *U. pinnatifida* grows range from 3.5 to 20 °C (Floc’h et al., 1991), very similar to Irish conditions of 6 to 18 °C (Marine Institute 2016). Reproduction takes place between 7 and 23 °C (Sanderson 1990), which also corresponds with Irish water temperatures to allow for growth and maturity of gametophytes. Taking into account that boating traffic regularly crosses between Ireland, England and France, it is conceivable that *U. pinnatifida* may expand its range to include the Republic of Ireland.

Kraan (2008) undertook large-scale surveys in 2000 and 2001 at the south East coast of Ireland including SCUBA checks. Several high boating traffic marinas were checked as these marinas harbour traffic between Ireland, England, France and hence were prime candidates for early colonization of *Undaria*. Pontoons and boat hulls (areas of low competition which may provide an easy structure to colonize) were also searched; however, they did not show indication that *Undaria* had established in Irish waters. We did however report another invasive brown seaweed species for the first time in Ireland, *Sargassum muticum* (Yendo) Fensholt (Kraan 2008).

**Fig. 1:**
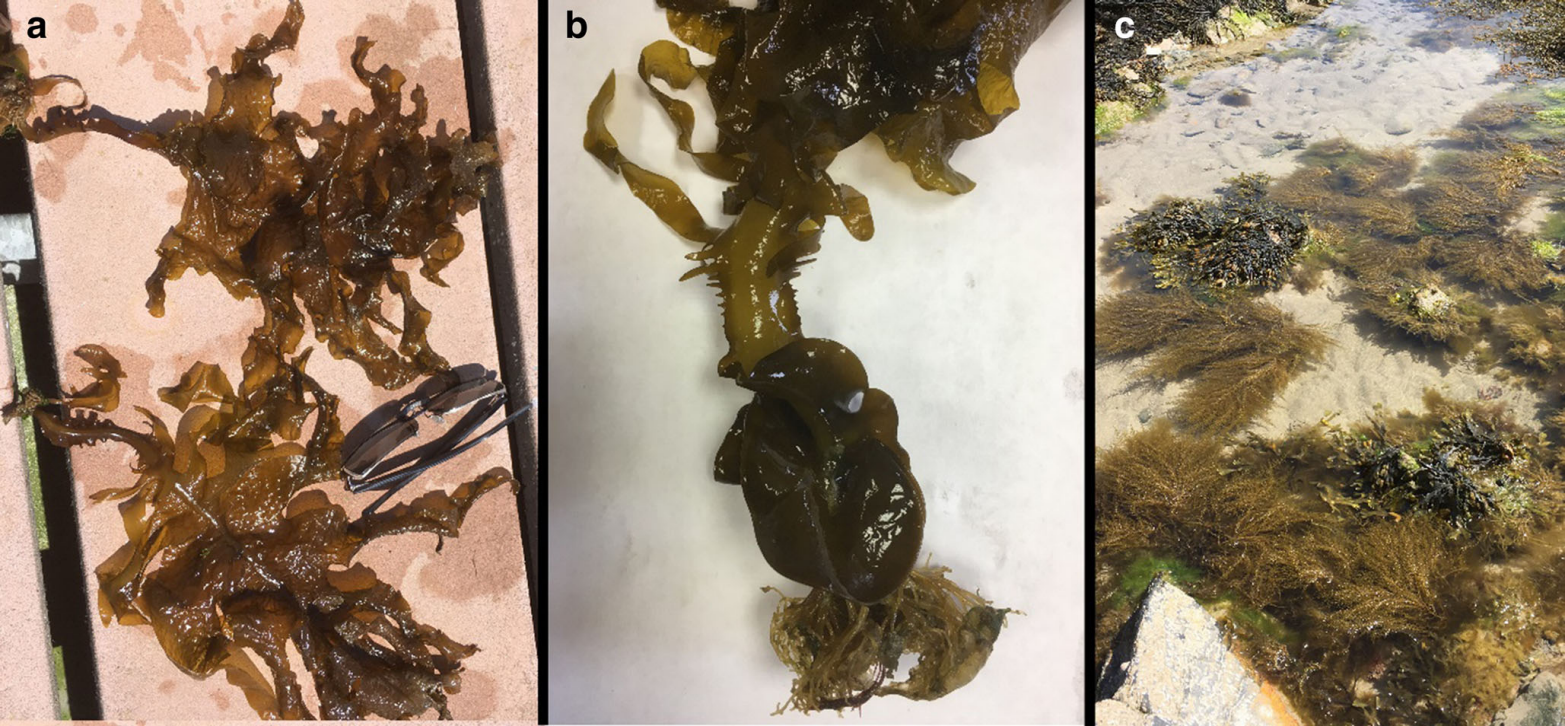
**a** *Undaria pinnatifida* collected from a pontoon at Kilmore Quay, County Waterford, Republic of Ireland; *sunglasses* are for size reference. **b** Developing sporophylls on freshly collected *Undaria pinnatifida*. **c** Rockpools in the high intertidal with *Sargassum muticum* plants. All collected specimens are pressed and stored at the Ocean Harvest Technology Herbarium

The marina at Kilmore Quay, Co. Wexford (52.1757° N, 6.5864°) was revisited in June 2016 after a gap of 15 years. *Undaria* was found on all the pontoons of the commercial, as well as the yachting part, of the marina showing a resident population (Fig. 1a, b). Many fertile plants were also found, indicating that a steady population has been present for several years at least.

## Ecological impact

*Undaria* has caused changes to the composition and cover of native macroalgal communities (Curiel et al. 1998; Valentine and Johnson 2003). Manual removal reduces sporophyte numbers; however, in established populations, survival over 2.5 years can occur due to microscopic small gametophytes which survive in microhabitats (Hewitt et al. 2005). Moreover, small sporophytes late in the season form mature sporophylls within just 1 month (Schaffelke et al. 2006) increasing the population’s survival. The eradication, or even control, of marine invasive species is both technically difficult and costly (Zavaleta et al. 2001; Lovell et al. 2006). Globally, few marine incursions have been eradicated successfully (Wotton et al. 2004).

At the French Mediterranean coast, *Undaria* mostly co-occurs with species of the brown algal genera *Sargassum* C. Agardh, *Cystoseira* C. Agardh and *Dictyota* J.V.Lamouroux and with the red algal genus *Gracilaria* Greville without negative effects on composition and cover of other macroalgae (Boudouresque et al., 1985). Twelve years after introduction at the French Atlantic coast, Floc’h et al. (1996) commented that it was difficult to recolonise a denuded rocky area in an in situ experiment and that *Undaria* cannot compete with local kelps, especially the opportunistic *Saccorhiza polyschides* (Lightfoot) Batters. Four years after its introduction in southern England, it occurs mainly on vertical sides of floating structures, such as pontoons, hulls of small boats, buoys, ropes and tyres. In the Torquay marina, however, it grows also on fixed objects and has colonized walls, where native kelps such as *Laminaria digitata* (Hudson) J.V.Lamouroux and *Saccharina latissima* (Linnaeus) C.E.Lane, C. Mayes, Druehl & G.W.Saunders occur, and also co-occurs in places with the annual kelp *S. polyschides*, scattered fucoids, and the introduced Japanese brown alga *S. muticum* (Fletcher and Farrell, 1999). They stated that it is more common than native species in sheltered and turbid areas and that, in more exposed areas, the competition from native canopy species is quite high.

On the English south coast, Fletcher and Farrell (1999) did not report any ecological impact on native seaweeds, even when occurring together in some areas. On the whole, they concluded that *Undaria* will establish itself mainly in the shallow sublittoral zone, though it has little competitive ability there. Nevertheless, it probably will stay as one of the major fouling algae in harbour areas. They also pointed out that, because *Undaria* grows even in areas with high sediment load and lower salinities where less native vegetation occurs, it may even be beneficial to the ecosystem by providing a nursery ground for small fish and shelter for macrofauna. Later studies have shown that *Undaria* has outcompeted some native species in a marina (Farrell and Fletcher 2006). Along the Galician coast, two decades after its introduction, all of the available evidence indicates that this Asian kelp has no appreciable impact because it occupies “empty” niches or disturbed communities (Peteiro et al. 2016). These authors reported that the Spanish Government has enacted invasive alien species legislation, but *U. pinnatifida* was not included as an invasive or potentially invasive species.

Surveying the marina in Kilmore Quay and the surrounding rocky shores and beaches (June 2016), our observations agree with the observations of Fletcher and Farrell (1999) and Peteiro et al. (2016). No noticeable ecological impact on native seaweeds was observed. As a matter of fact, no plants were found outside the harbour while the *Undaria* population has been present in the marina for at least several years. This is in contrast to *S. muticum* of which a large population had established itself on the rocky shores from high intertidal pools to the subtidal during the survey in 2001 (Kraan 2008). In July 2016, however, *S. muticum* was only present in a few rock pools in the high tidal zone and its dominant cover in 2011 had completely disappeared to sporadic appearances in rock pools. In the case of *Sargassum*, it seems to have found its own niche in the intertidal, mainly as part of the rock pool macroalgae assemblage (Fig. 1c). It seems that *Undaria* is one of the dominant fouling organisms in harbours and marinas and other manmade structures like mussel lines (Castric-Fey et al. 1993) but so far has not invaded the subtidal or lower intertidal in Kilmore Quay.

## Control and management

Gametophytes are very tolerant to light and temperature fluctuation and are able to survive in the dark for long periods of time (Tom Dieck 1993). Hay (1990) demonstrated that sporophytes can survive while growing on the hulls of ships for voyages over 4000 km, making it nearly impossible to stop spreading of the species. Gametophytes can survive high temperatures around 30 °C for up to 10–40 days (Kim and Nam 1997), indicating that boat hulls need high temperature treatment to eradicate the gametophytes. Gametophytes can easily survive in moist spaces in structures of the boat hull, facilitating survival in dry docks and land transport to other harbours. Ballast water is another possibility, as gametophytes can survive darkness for an extended amount of time (Tom Dieck 1993). Treatment with UV or high temperature is the only way to prevent spreading not only of *Undaria* but many other species as well. When *Undaria* was first recorded on the English south coasts, all plants found were removed, but because the plants were already fertile, eradication failed, and new plants appeared (Fletcher and Farrell, 1999).

A study by Hewitt et al. (2005) demonstrated that 2.5 years after sporophyte removal and continuous monitoring, the species would grow back due to an established seed bank of gametophytes in the substratum. Eradication of established populations of *Undaria* is virtually impossible due to the plant’s reproductive characteristics and the current lack of effective eradication techniques. Eradication may be possible with early detection of a new infestation (i.e. prior to spore release) that is confined to a small area, e.g. in New Zealand when a fishing vessel with *U. pinnatifida* on the hull sank near the Chatham Islands in 2000, hot water sterilization was then used to achieve successful eradication (Wotton et al. 2004). In general, eradication programmes often fail as one missed plant can re-infest an area and return a monitoring and eradication programme back to the starting point. With global trade and movement of freight as well as people, the question that remains thus is if we should try to control and manage these invasive species in the first place if eradication and control are expensive exercises and often fail (Zavaleta et al. 2001; Lovell et al., 2006).

## Cultivation and economic implications in Asia

Cultivation of *U. pinnatifida* started in Dalian, China, before and during World War II. After 1955, cultivation started in earnest by Japanese fisherman of the Sanriku and Naruto areas. Large-scale cultivation began in China in the mid-1980s due to increasing demand by Japan. Currently, the two Northern provinces, Liaoning and Shandong, are the main production areas for *U. pinnatifida* (McHugh 2003). From a total area of 7693 ha in 2014, China harvested 203,099 t dry weight of *Undaria*, corresponding to 2,030,990 t wet weight in FAO statistics contributing well over 900 million US$ (FAO 2016).

In Japan, there has been extensive cultivation at a commercial level since the mid-1950s (Yamanaka & Akiyama 1993). The development of salting technology increased the annual production from 50,000 to 120,000 t (McHugh, 2003); however, in 2014, Japan harvested a total of 43,900 t wet weight (FAO 2016). Recent work on the breeding of an elite cultivar of *U. pinnatifida* through gametophyte clone crossing and consecutive selection has improved yields by 40 % to 20 kg m^−1^ of rope while providing wider mid ribs and blades as is desired by the commercial industry (Shan et al. 2016). As of 2014, nearly 50 % of the Chinese production of *Undaria* was exported to the Japanese markets.

In the Republic of Korea, rope cultivation of *U. pinnatifida* began in 1964, and subsequently, the cultivation was largely developed, promoted and industrialized in the 1970s. Most of the production is produced from Wando County in Jeonnam Province south of the country. The 2014 harvest was 283,707 t accounting for close to 30 % of total seaweed farming production in Korea with a value of 67.5 million US$. Apart from being a popular seaweed species for human consumption in the Republic of Korea, it is also farmed as feed for the cultivation of abalone, of which most production operations using marine cages also occur in Wando County (FAO 2016).

## Products

The quality of *Undaria* products is mainly valued on its thickness and hardness that is determined by cultivation and processing conditions including dehydrated or dried, seasoned and instant Wakame food (fresh green being the most preferable to people; Yamanaka and Akiyama 1993). Other factors of importance are storage quality (such as stability of pigment or elasticity) and the absence of epiphytes and sand. Dried-cut Wakame is prevalently consumed in market as an instant processed food (Chritchley and Ohno 1998).

Moreover, the dried products retain a nice fragrance with moderate elasticity for a long period during storage. Further techniques for Wakame processing have been developed, including salted, boiled-salted and dried-cut Wakame products. Wakame products can be classified as three main product forms, including the frozen sporophylls, cooked and salted blades and midribs. These three forms are further processed into many other kinds of food and snack products (Abbott 1990; Druehl 1990). The dry raw material value of the current Wakame harvest is approx. US$ 1 billion (FAO 2016). The Wakame food and snack industry represent a multiple value of this figure which makes this an interesting species in respect of economic value.

## Cultivation and economic implications in Europe

*Undaria pinnatifida* was deliberately introduced in Brittany in 1983 for commercial cultivation and exploitation and initially cultivated at three locations producing about 8 t dry weight a year (Werner et al. 2004). French authorities now limit the farming of *U. pinnatifida* in those areas where it has been cultivated for a long time or where it forms dense stands, and farming is always under strict control to prevent potential ecological impacts and further spread. At least one company cultivates *U. pinnatifida* in northern France (Aquaculture, 2016). Small-scale cultivation also took place in Galicia Northern Spain since 1997. Currently, commercial cultivation of *U. pinnatifida* is being developed in Northwest Spain (Peteiro and Freire 2012). Though it is now being cultivated on a small commercial scale in waters off the Brittany coast and the Galician coast, almost all commercial cultivated *U. pinnatifida* comes from Asia. New cultivars with properties such as high yield or high environmental adaptability have been developed in China (Shan et al. 2016).

The standard yields obtained for *Undaria* are around 10 kg fresh m^−1^ rope (Peteiro et al. 2016; Shan et al. 2016) and around 16 kg fresh m^−1^ rope for *S. latissima* cultivation (Peteiro and Freire 2013). Yields for *Undaria* compare to ranges reported for commercial farms in their native Asian waters (Akiyama and Kurogi 1982). Cultivation at the southern distribution limit of *S. latissima* allows to obtain higher yields (almost double the biomass) compared with those reported in colder waters along the optimal distribution range of this species in the North Atlantic ocean (Buck and Buchholz 2004; Sanderson et al. 2012) and performing better than *Undaria*. In that case, a high-yield commercial kelp species might be of higher interest and might be preferential than an invasive species for food and biorefinery applications (van den Burg et al., 2016). On the other hand, high levels of mannitol in *S. latissima* (Kraan 2012) makes the species less suitable for animal feed as it can induce diarrhoea as mannitol attracts water from the intestinal wall (Palacios et al. 1987).

## *Undaria* and health benefits

Several reviews have shown that *Undaria* as whole food, as extract, or compounds extracted from *Undaria* has demonstrated antibiotic, mitogenic, anti-tumour, anti-proliferation, cytotoxic, anti-inflammatory, anti-adhesion, anti-human immunodeficiency virus (anti-HIV) activity, ACE inhibitory, anti-oxidant and human platelet aggregation inhibition (See Smit 2004; Holdt and Kraan 2011; Se Kwon Kim 2012; Taboada et al. 2013). In view of the increase of obesity and degenerative diseases in our population, there are a few studies highlighted here in respect of *Undaria* consumption and possible health benefits. Maeda et al. (2005) demonstrated that fucoxanthin from Wakame can help burn fatty tissue. Studies in mice have shown that fucoxanthin induces expression of the fat-burning protein UCP1 that accumulates in fat tissue around the internal organs. Expression of UCP1 protein was significantly increased in mice fed fucoxanthin. A study by (Wang et al. 2014) showed the inhibitory effect of pure fucoxanthin and extract from *Undaria* on nine different cancer cell lines. Overall, fucoxanthin is effective in inhibiting the growth of lung carcinoma, colon adenocarcinoma and neuroblastoma; however, better results were obtained using an extract than the pure compound. This may be due to a combination of other bioactive polysaccharides like fucoidan (Atashrazm et al., 2015). Wijesekara et al. (2011) reported on the effects of fucoidan from *Undaria* and the effect against *Herpes simplex* virus, and the induced osteoblastic cell differentiation which might play a role in bone health. Atashrazm et al., 2015 reported on the positive effects of fucoidan from *Undaria* against several types of cancer. Results suggest that extracts with fucoxanthin and fucoidan may be used as a chemo preventive phytochemicals or in combination chemotherapy (Wang et al. 2014; Atashrazm et al., 2015). Sulfated polysaccharides from *S. latissima* have similar activities (Croci et al. 2011; Ehrig and Alban 2015).

## Discussion

Marine invasive macroalgae can establish in communities because they are better competitors than native species; however, in order to remain, the competitive advantage must be persistent over time. Factors such as population size, gene flow, genetic drift, genetic diversity, fitness and coevolution all play a role in the ability of native or invasive species to evolve competitive ability against one another (Leger and Espeland 2010). If the genetic diversity of the invasive and native species is at a similar level, it will result in that both species will co-exist and the invasive species will establish itself in the native algal flora. In the case of the invasive alga *S. muticum*, we have observed a strong increase in growth and settlement all around the isle of Ireland since it was first discovered in Ireland (Kraan 2008). However, after 15 years, it seems to have established as one of the native macroalgae with under certain conditions some dominance in mid-intertidal rock pools. When does an invasive species become part of the local flora and is considered a native species? A question that up to date has not properly been addressed, but will most probably be based on a historical time frame or reference. For example, the red alga *Asparagopsis armata* Harvey was first discovered in the British Isles 1939 in Galway Bay, Ireland by De Valéra (1939, 1942). Less than 60 years later, the species has been cultivated for commercial purposes in the mid-1990s in Ard Bay in Ireland for cosmetic applications (Kraan and Barrington 2005). The questions remain if *Undaria* cultivation in Europe should be allowed or not. The French authorities have limited the farming of *U. pinnatifida* in those areas where it has been cultivated for a long time or where it forms dense stands. Farming is always under strict control to prevent ecological impacts and further spread, however that seems a rather pointless statement after allowing introduction and farming for 33 years. In New Zealand, *Undaria* is classified as an unwanted organism under the Biosecurity Act 1993 and is also listed under the regional pest management strategy of some regional councils. In contrast, a revised policy was introduced in 2010 which does allow farming of *Undaria* in heavily infested areas and harvest when it grows on artificial surfaces (Ministry for Primary Industries 2016). This follows in the footsteps of the French example were on the one hand the Government tries to prevent introduction and spreading of an invasive unwanted species, however on the other hand, sees the economic potential allowing farming and harvest (Ministry for Primary Industries 2016). In Spain, *Undaria* does not even feature on the invasive species list (Peteiro et al. 2016). By allowing farming in the north Atlantic and the cross channel boating traffic, *Undaria* will form, or already has formed, part of our biodiversity in Ireland, Northern Ireland, Great Britain, France and Spain and will most probably further expand its range of distribution. Of more interest is to see how far the species will be able to spread north and southwards. Therefore, objection to farming would not make sense. There is also no argument for environmental or ecological impacts as shown by Fletcher and Farrell (1999) and Peteiro et al. (2016).

From a biomass and weight perspective, *Undaria* performs just as well as *S. latissima* with both species able to reach about 20 kg m^−1^ rope ( Peteiro et al. 2016; Shan et al. 2016).

Hence, the only consideration that plays a role is an economic one. In that case, taking into account the large body of peer reviewed evidence on human health aspects, the numerous bioactives in *Undaria* coupled to a readily available market for sea vegetables and snacks, makes *Undaria* the species of choice to farm.
